# Clinical, social, molecular, and genetic predictors of cognitive resilience in long-living adults without dementia

**DOI:** 10.3389/frdem.2025.1699695

**Published:** 2026-02-16

**Authors:** Ekaterina Spektor, Aleksandra Mamchur, Mariia Bruttan, Liliya Artemieva, Antonina Rumyantseva, Lorena Matkava, Mikhail Ivanov, Veronika Daniel, Sergey Igorevich Mitrofanov, Irina Strazhesko, Vladimir Yudin, Valentin Makarov, Anton Keskinov, Olga Tkacheva, Daria Kashtanova, Sergey Yudin, Veronika Skvortsova

**Affiliations:** 1 Federal State Budgetary Institution «Centre for Strategic Planning and Management of Biomedical Health Risks» of the Federal Medical and Biological Agency, Moscow, Russia; 2 Separate Structural Unit “Russian Gerontology Research and Clinical Centre” of Pirogov Russian National Research Medical University of the Ministry of Health of the Russian Federation, Moscow, Russia; 3 The Federal Medical Biological Agency (FMBA), Moscow, Russia

**Keywords:** aging, brain maintenance, cerebral reserve, cognitive impairment, cognitive reserve, cognitive resilience, dementia, long-living adults

## Abstract

**Background:**

Long-living adults often maintain cognitive function despite neuropathological changes, which is often attributed to cognitive resilience (CR)—a combined effect of cognitive and cerebral reserves. CR is influenced by genetic, clinical, sociodemographic, and environmental factors.

**Materials and methods:**

We investigated genetic, clinical, and environmental predictors of CR in 198 dementia-free long-living adults via two neuropsychological examinations over a 2-year period, a geriatric assessment, and a genome-wide association study (GWAS).

**Results:**

Limited mobility, reduced walking, hearing impairment, depression, anemia, lower quality of life, and decreased BMI were key accelerators of CI. Depression, hypercholesterolemia, and lack of hobbies increased the risk of mild cognitive impairment (MCI)-to-dementia progression. GWAS identified CR-associated genetic variants, including a missense mutation in *SYNGAP1* (Ile1115Thr) not previously linked to cognitive disorders.

**Conclusion:**

Our findings corroborated established risk factors for cardiovascular diseases and identified population-specific patterns, with APOE ε4 showing no significant association. Both protein-coding regions and non-coding elements were implicated in CI, suggesting that it is underlain by complex regulatory mechanisms.

## 1 Background

Aging leads to dendritic shortening and reduced arborization. The resulting disruption of neurotransmission and decrease in synaptic density (Kaur and Rattan, 2024) contribute to neurodegeneration and impaired memory and learning (Gaspar-Silva et al., 2023). The risk of late-onset Alzheimer’s disease (AD) doubles every 5 years between 65 and 85 years of age, but this trend may plateau or reverse after the age of 90 (Kawas and Corrada, 2006). Key midlife risk factors, such as APOE ε4, cardiovascular and metabolic disorders, etc., tend to diminish in very advanced age (Kawas and Corrada, 2006; Livingston et al., 2020), highlighting the unique traits of this demographic.

Neuropathology, such as amyloid plaques, tauopathy, and brain shrinkage, is common in long-living adults (Elobeid et al., 2016). However, its clinical manifestations vary significantly: 50% of long-living adults with dementia do not exhibit clear neuropathological correlates, while 33% of cognitively healthy long-living adults meet AD criteria (Polvikoski et al., 2001; Katzman et al., 1988; Crystal et al., 1988).

The concept of cognitive reserve, a relatively recent development in modern neurobiology, seeks to explain the observed discrepancy between the extent of brain damage caused by various pathological processes and the severity of associated cognitive impairment (Stern, 2009). Individuals with a higher cognitive reserve tend to be at a reduced risk of developing dementia and experience a slower progression of the existing cognitive decline. This advantage is attributed to enhanced brain function, supported by a greater number of neural pathways, allowing for the utilization of different cognitive strategies for problem-solving.

Cognitive reserve comprises both passive (cerebral) and active components. The passive component reflects the brain’s structure, such as volume and synaptic density, that determines the ability to withstand pathological changes (Stern et al., 2019; Koberskaya and Tabeeva, 2019; Perneczky et al., 2010). The active component involves adaptive neural processes—efficient use of existing networks and recruitment of alternative pathways—to compensate for damage (Stern, 2002). These passive and active mechanisms, together with brain maintenance and neurocognitive compensation, constitute cognitive resilience (CR) (Stern et al., 2019; Kochhann et al., 2023; Cherdak, 2023), which accounts for the varying extent of cognitive decline among individuals affected by similar pathological processes.

Several projects have examined factors influencing cognitive status in the elderly and oldest-old. For instance, the Nun Study of Aging and Alzheimer’s Disease demonstrated that higher educational attainment and greater early-life linguistic ability support CR, whereas the presence of the APOE ε4 allele undermines it (Clarke et al., 2025). Although the 90 + Study did not focus on cognitive dynamics, its findings suggest no association between dementia prevalence in adults aged 90 + years and various factors, including estrogen therapy, BMI, vitamin A, C, and E supplementation, total vitamin A intake, alcohol or caffeine consumption, and physical activity levels (Kawas, 2008). The findings of the Lothian Birth Cohort studies suggest that while cognitive ability in early life does not ensure cognitive resilience, lower physical activity and the presence of the APOE ε4 allele may contribute to greater cognitive decline (Corley et al., 2018).

There is no single approach to assessing cognitive reserve, or CR. The existing scales (Nogueira et al., 2022) focus either on socio-demographic factors and lifestyle or neuropsychological correlates of intellectual abilities. This involves comprehensive neuropsychological testing and the assessment of the pre-decline level of education attainment and lifelong intellectual engagement. From a practical perspective, it is crucial to evaluate individual cognitive resilience—defined as the ability to maintain the current level of cognitive functioning. Based on theoretical models that define CR as a product of innate predisposition and life-course factors, we hypothesized that CR in long-living individuals it would be predicted by a combination of genetic and sociodemographic factors.

## 2 Methods

### 2.1 Participants

This study is part of a collaborative research initiative to identify factors contributing to healthy longevity, undertaken by the Centre for Strategic Planning and Management of Biomedical Health Risks and the Russian Gerontology Research and Clinical Centre. To ensure complete datasets for analyses, only participants who underwent cognitive evaluation using the Mini-Mental State Examination (MMSE) at recruitment (2019–2022) and follow-up (2022–2024) were included in the study. The exclusion criteria were dementia at recruitment [MMSE < 25 points (Lewis and Trempe, 2017)], debilitating somatic disorders, Parkinson’s disease, active cancer, acute cerebrovascular accident (ACA), and traumatic brain injury.

Participants underwent a comprehensive assessment including the analysis of medical history and socioeconomic background (education, living arrangements, social interactions, mobility, smoking status, employment/income, health conditions, and medications), physical examination (anthropometrics, blood pressure, and heart rate), cognitive screening test (MMSE) (Upton, 2013) with amnestic/non-amnestic classification (Anand and Schoo, 2024), mental health evaluation [the five-item version of the Geriatric Depression Scale, GDS-5, ≥2 for depression (Hoyl et al., 1999)], and quality of life rating (10-point scale: 0 = worst, 10 = best). Based on their MMSE scores, participants were categorized as having normal cognition, mild cognitive impairment (MCI), or dementia (corresponding to scores of >27, 25–27, and <25, respectively).

### 2.2 Laboratory analyses, DNA sequencing, and bioinformatics

Blood samples were analyzed for hematological parameters (white/red blood cells, platelets, hemoglobin, and ESR), biochemical markers (glucose, bilirubin, total protein, albumin, GGT, creatinine, urea, HbA1c, and apolipoprotein A1), lipid profile (total cholesterol, HDL, LDL, and atherogenicity index), liver enzymes (ALT and AST), and hormonal levels (TSH, vitamin D, B12, adiponectin, leptin, and insulin) using standard methods, including photometry, the biuret test, colorimetry, capillary electrophoresis, immunoturbidimetry, enzymatic assays, and chemiluminescence.

DNA was extracted from whole blood samples using the QIAamp DNA Mini Kit (Qiagen, Germany). Whole genome sequencing libraries were prepared using the Nextera DNA Flex kit (Illumina, USA). The samples were sequenced to 150 bp reads using the NovaSeq 6000 S4 Reagent Kit (300 cycles) (Illumina, USA).

FASTQ files were obtained by demultiplexing the sequencing data in BCL format using the bcl2fastq2 Conversion Software v2.20 (Illumina, 2017). Flow cell-wide sequencing quality was assessed using Sequencing Analysis Viewer v2.4.7 (Illumina, 2024). The quality of the FASTQ files was evaluated using FastQC v0.11.9 (Babraham Bioinformatics, n.d.). The reads were aligned to the reference genome, GRCh38.d1.vd1 (GDC Reference Files, n.d.), using the Illumina DRAGEN Bio-IT Platform (v07.021.510.3.5.7) (DRAGEN Secondary Analysis, 2024). The alignment quality of the BAM files was checked using DRAGEN FastQC v0.11.9 (Babraham Bioinformatics, n.d.), SAMtools v1.13 (Li et al., 2009), and mosdepth v0.3.1 (Pedersen and Quinlan, 2018). All samples were checked for duplicates, unmapped reads, and other quality metrics. The mean sequencing coverage was at least 30x for all samples.

Small variant calling of up to 50 b.p. was performed using Strelka2 v2.9.10 (Illumina) (Kim et al., 2018). CrosscheckFingerprints (Picard), containing a haplotype map file (Najafov and Najafov, 2017), was used to check for duplicates. The bioinformatics pipeline was validated using the HG001 reference genome from the Genome in a Bottle (GIAB) consortium (v.3.3.2) (F-score = 99.83%) (Krusche et al., 2019).

### 2.3 Data analysis

Statistical analyses were performed in R (RStudio 2024.09.1 + 394) (R: The R project for statistical computing, 2024). Continuous variables were presented as medians and interquartile ranges; categorical variables, as counts and proportions. For group comparisons, the Mann–Whitney *U* test was used for continuous variables, and Pearson’s chi-square test was used for categorical variables. Regression modeling was employed to identify predictors of MCI-to-dementia progression.

Multiple linear regression was used to identify predictors of cognitive impairment progression, with CR as the primary endpoint. CR was calculated as: (follow-up MMSE / initial MMSE) × 100%, adjusted for the duration of the assessment interval. Initial models included sex, age, and baseline MMSE as covariates. Significant predictors (*p* < 0.05) were selected via backward elimination using F-tests and used in the final model. Logistic regression was used to analyze MCI-to-dementia progression in MCI participants, with dementia diagnosis during the follow-up as the outcome. Each potential risk factor was evaluated, accounting for sex, age, and the assessment interval. Significant predictors (*p* < 0.05) were included in the final model following backward elimination via likelihood-ratio tests.

### 2.4 Genome-wide association study and protein modeling

To account for population structure, the first 10 principal components obtained in the principal component analysis (PCA) of the genomic data were included as covariates in the genome-wide association study (GWAS). For the purpose of cross-validating the GWAS results, a five-fold random selection of sub-cohorts was carried out, encompassing 80% of the entire cohort. Separate GWASs were conducted for each sub-cohort.

Variants violating the Hardy–Weinberg equilibrium (*p*-value < 10^−6^); variants with an allele frequency (AF) of more than 0.98; multiallelic variants; and variants with a minor allele frequency (MAF) of less than 0.01 were removed from the GWAS.

Genome-wide associations were tested using the following linear regression equation:


Y=β0+βc∗C+βg∗G

where, *𝛽_0_* = constant, *𝛽_c_* = covariate effect vector, *C* = covariate vector, *𝛽_g_* = genotype effect vector, *G* = genotype vector.

Calculations were performed using statsmodels v0.12.2 in Python and parallelized in Spark Cluster. Covariates included age, sex, and educational attainment as an ordinal variable (0, secondary education or lower; 1, secondary and vocational education; 2, higher education, including academic degrees), as well as the first 10 principal components that accounted for population structure. To average the GWAS results, the geometric mean was calculated for *p*-values, and the arithmetic mean was calculated for regression coefficients. Variants with an average *p*-value below 5.0 × 10^−8^ were considered to reach genome-wide significance.

Synaptic GTPase-activating protein (SynGAP), encoded by the SYNGAP1 gene, is a known trimer (Gamache et al., 2020). The amino acid sequence of the monomer was obtained from the UniProt database (UniProt, 2025) A three-dimensional full-atom model of the trimeric protein was created using the AlphaFold3 algorithm (Abramson et al., 2024) (Supplementary Figure S1) and visualized in the PyMOL system (The PyMOL Molecular Graphics System, 2025).

### 2.5 Polygenic risk modeling

The polygenic risk model incorporated GWAS-significant and sub-significant polymorphisms (*p* < 5 × 10^−7^) along with non-genetic predictors (baseline MMSE, GDS-5, pet ownership, hearing, mobility, walking frequency, quality of life, BMI, anemia, and cholesterol/glucose levels), adjusted for age and sex. Continuous variables were standardized using the mean values in the training set ± SD: age (91.96 ± 2.06), MMSE (25.63 ± 2.67), BMI (23.90 ± 8.77), glucose (5.42 ± 1.54), and cholesterol (4.92 ± 1.30).

PCA was carried out for dimensionality reduction, followed by exhaustive hyperparameter optimization (5–55 projections, *λ* = 10^−3^–10^3^). The ridge regression model (Python 3.10/scikit-learn 1.2.1) minimized:


$$\text{loss}=\sum_{i}{\left(y_{i}-\hat{y_{i}}\right)}^{2}+\lambda \sum_{j}{\beta_{j}}^{2}$$

where loss is the loss function to be minimized during training; y is the observed values; $\hat{y}$ is the predicted values (standardized using training parameters: *μ* = 96.96, SE = 10.04); *β* is the model coefficients; and λ is the regularization parameter. Final coefficients were obtained by reverse-transforming principal components to original variables.

## 3 Results

### 3.1 Cohort description

The study included 198 long-living adults with the median age of 91 years [90; 93]; 82.8% of them were women. Supplementary Table S1 provides a detailed overview of the cohort. The follow-up period continued for 2.0 years [1.8; 2.4]. Overall, participants exhibited high social engagement (83% interacted weekly; 72% regularly left home) and prevalent cardiovascular comorbidities: hypertension (90%), heart failure (60%), carotid atherosclerosis (17%), diabetes (14%), and stroke history (15%). Metabolic syndrome, polypharmacy, urinary incontinence, and chronic pain were present in 25, 47, 59, and 63% of the participants, respectively.

The median MMSE score was 27 (Babraham Bioinformatics, n.d.; Li et al., 2009) points at the enrollment and 27 (Hoyl et al., 1999; DRAGEN Secondary Analysis, 2024) points at the end of the follow-up period. At enrollment, 42% (*n* = 84) showed no impairment (MMSE>27), while 58% (n = 114) had MCI (MMSE 25–27), including 25% (*n* = 28) with the amnestic subtype. The median CR was 98.2% [94.0–101.4]. During the follow-up, 43% of the participants maintained stable MMSE scores, and 19% showed minimal changes (1–2 points). Dementia developed in 17% of cognitively healthy participants and in 45% of MCI patients.

#### 3.1.1 Clinical, social, and laboratory predictors of cognitive resilience

The analysis of sociodemographic, lifestyle, and clinical factors revealed several significant predictors of CR. In adjusted linear regression models, limited mobility (bedridden: *β* = −10.2, SE 3.3, *p* = 2.5 × 10^−3^; indoor-only: *β* = −4.1, SE 1.4, *p* = 5.3 × 10^−3^), hearing loss (*β* = −4.5, SE 1.6, *p* = 5.4 × 10^−3^), depression (*β* = −2.8, SE 1.2, *p* = 2.2 × 10^−2^), and chronic anemia (*β* = −3.7, SE 1.7, *p* = 3.5 × 10^−2^) were associated with greater cognitive decline. Protective factors included walking (*β* = 5.8, SE 1.6, *p* = 3.3 × 10^−4^), pet ownership (*β* = 3.3, SE 1.6, *p* = 3.9 × 10^−2^), higher BMI (*β* = 0.43, SE 0.16, *p* = 6.6 × 10–3), and better self-rated quality of life (*β* = 0.87, SE 0.3, *p* = 5.7 × 10^−3^). Among biochemical markers, only total cholesterol (*β* = −1.0, SE 0.51, *p* = 4.9 × 10^−2^) and glucose (*β* = −0.96, SE 0.47, *p* = 4.5 × 10^−2^) showed significant negative associations with CR. Low lifelong income (*β* = −5.2, SE 3.1, *p* = 9.9 × 10^−2^), total protein (*β* = 0.18, SE 0.11, *p* = 9.7 × 10^−2^), and TSH (*β* = −0.54, SE 0.21, *p* = 5.9 × 10^−2^) showed statistically meaningful trends.

**Figure 1:**
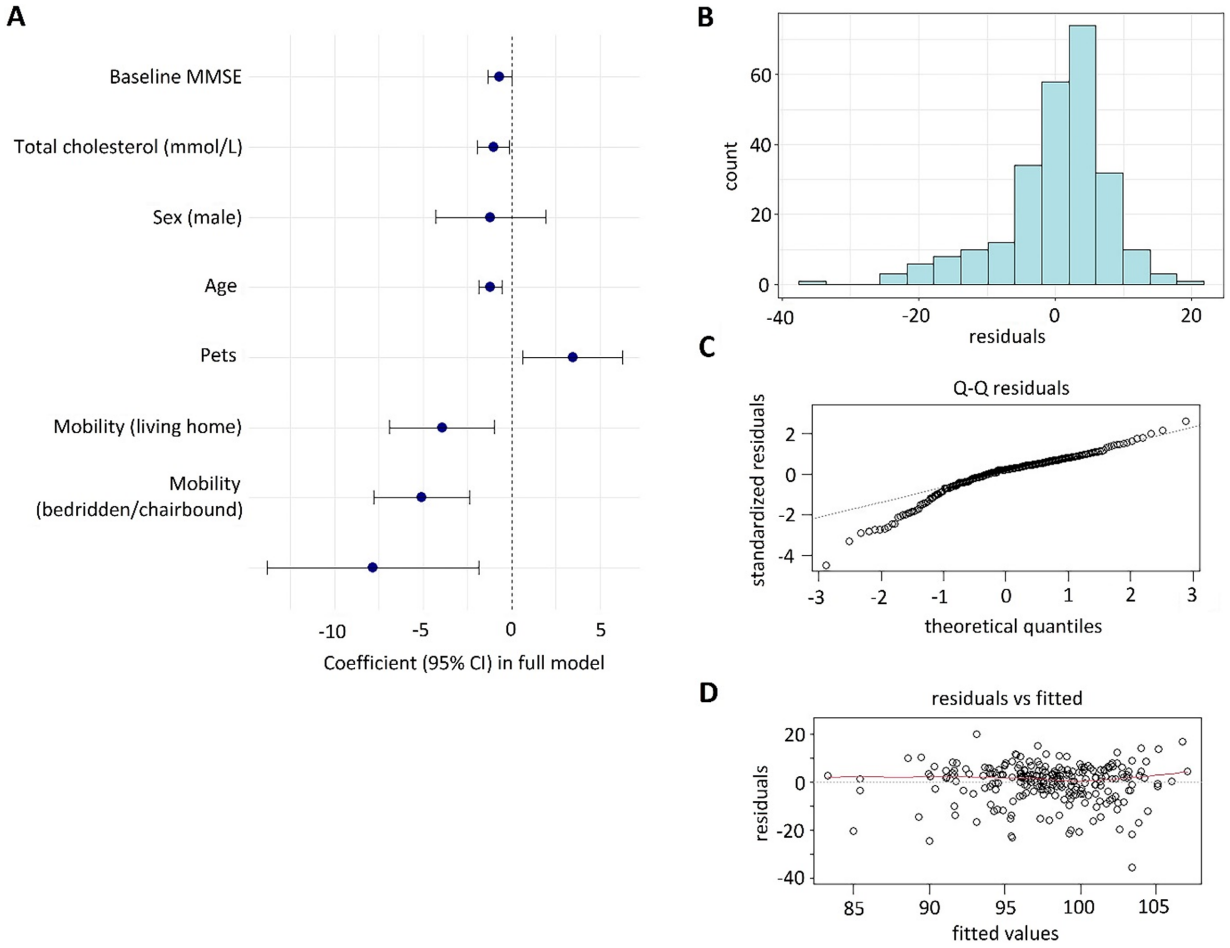
Characteristics of the regression model predicting the likelihood of maintaining the current cognitive status in long-living adults. **(A)** Coefficients and 95% CI for the predictors included in the final multiple linear regression model. **(B)** Distribution of model residuals. **(C)** Q-plot of the distribution of model residuals. **(D)** Dependence of residuals on the predicted values. Residuals, model residuals; number, number of observations; Q-Q residuals, Q-plot of the distribution of model residuals; standardized residuals: Standardized model residuals; residual vs. fitted: The relationship between residuals and predicted values; fitted values: Predicted values.

No significant associations were found for education, living arrangements, cardiovascular comorbidities, or vitamin D levels. Supplementary Table S2 presents the results of the regression analyses. In the final multiple regression model, several initially significant predictors (walking frequency, glucose, BMI, quality of life, and depression) lost significance after adjustment for other factors. The loss of significance is likely attributable to limited statistical power due to the cohort size and may reflect collinearity shared variance among predictors. The model residuals showed non-normal distribution (*p* < 0.001), indicating unaccounted variability in severe cognitive decline cases. Table 1 presents the parameters included in the final multiple linear regression model. Figure 1 illustrates the characteristics of the model and its performance indicators. The asymmetric distribution of residuals with a long tail of negative values (Figures 1B,C) indicates a subgroup of individuals with significant cognitive decline that our model could not explain. This cohort likely comprises individuals who developed progressive neurodegenerative conditions independent of the factors included in our analysis. Furthermore, the residuals demonstrate independence from predicted values and an absence of heteroscedasticity (Figure 1D).

**Table 1:** Parameters of the multivariate regression model assessing social, clinical, and laboratory factors affecting CR in long-living adults.

Parameter	Regression coefficient (standard error)	*t*-value	*p*-value
Intercept	232.2 (32.3)	7.18	1.8 × 10^−11^
Age, years	−1.2 (0.3)	−3.64	3.6 × 10^−4^
Sex, men	−1.2 (0.6)	−0.74	4.6 × 10^−2^
Initial MMSE score	−0.7 (0.3)	−1.98	4.9 × 10^−2^
Mobility, moving indoors*	−5.1 (1.3)	−3.70	2.9 × 10^−4^
Mobility (bedridden/chairbound)*	−7.9 (3.1)	−2.57	1.1 × 10^−2^
Anemia	−3.9 (1.5)	−2.58	1.1 × 10^−2^
Pet ownership	3.5 (1.4)	2.40	1.8 × 10^−2^
Cholesterol, mmol/L	−1.0 (0.5)	−2.24	2.6 × 10^−2^

*Three categories are distinguished within the ‘mobility’ parameter, with ‘leaving the home’ as the reference point.

#### 3.1.2 Factors of MCI-to-dementia progression

In the MCI subgroup (*n* = 114), logistic regression identified depression (*β* = 0.86, SE 0.41, *p* = 3.4 × 10^−2^), elevated total cholesterol (*β* = 0.44, SE 0.18, *p* = 1.2 × ^10–2^), higher LDL levels (*β* = 0.46, SE 0.21, *p* = 2.8 × 10^−2^), and cohabiting with family [vs. living alone (*β* = 0.87, SE 0.43, *p* = 4.0 × 10^−2^)] as significant risk factors for dementia progression. Having hobbies (*β* = −1.0, SE 0.45, *p* = 2.4 × 10^–2^) and higher scores on the subjective quality of life scale (*β* = −0.34, SE 0.12, *p* = 5.1 × 10^–3^) reduced the risk of MCI-to-dementia progression. No associations were found for education, cardiovascular comorbidities, sensory acuity, mobility, or socioeconomic factors. Supplementary Table S3 presents all results of the regression analysis of the dependent variable.

**Figure 2:**
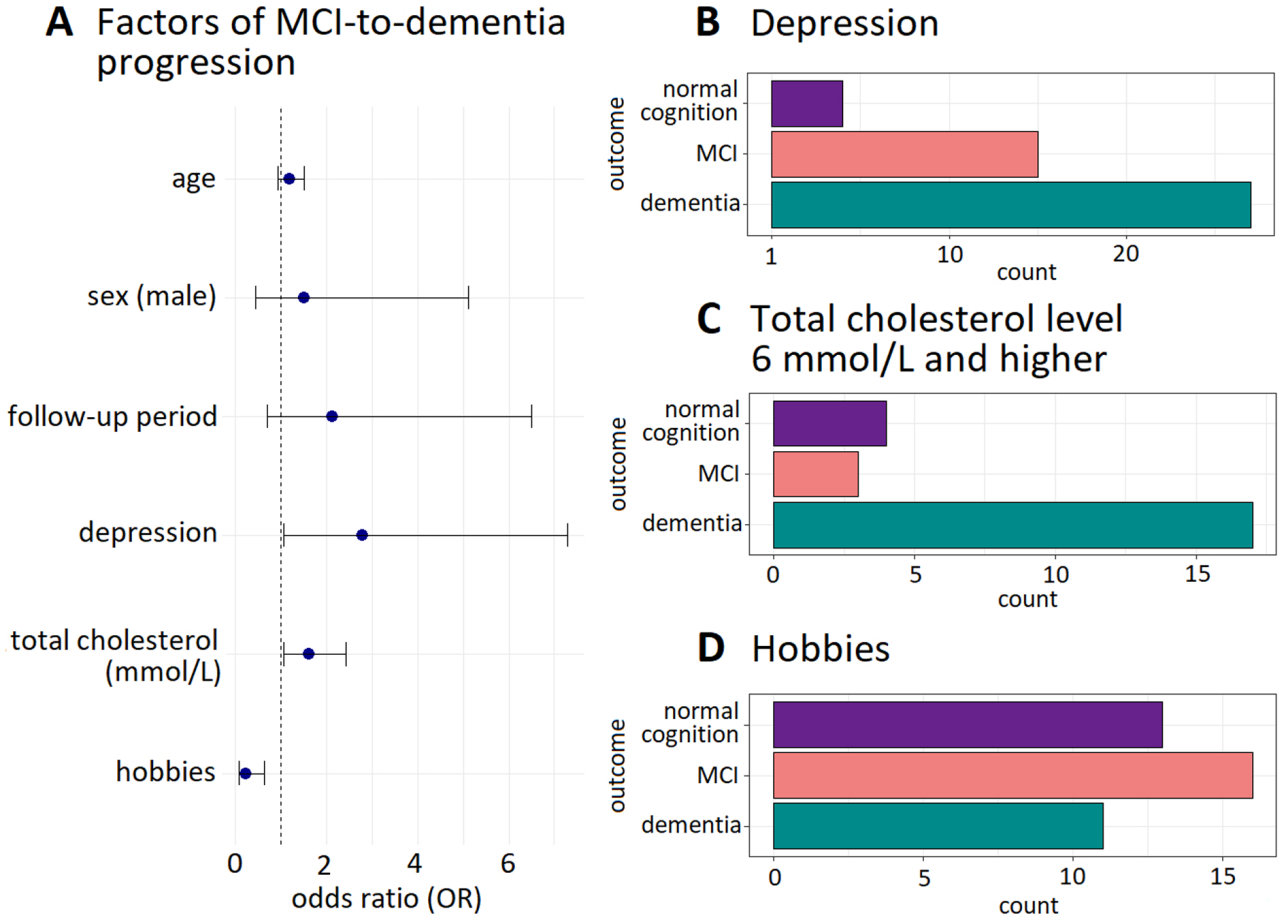
Predictors of MCI-to-dementia progression. **(A)** Odds ratio and the 95% CI for predictors included in the final multiple logistic regression model. **(B–D)** Cognitive outcomes in participants with depression, hypercholesterolemia, and hobbies, respectively.

The final multivariate model retained depression (58.7% progression rate), total cholesterol ≥6 mmol/L (70.8% progression), and hobby engagement (72.5% cognitive stability) as key predictors after excluding non-significant variables (living arrangements, LDL, quality of life). Model performance is detailed in Table 2 and Figure 2.

**Table 2:** Parameters of the multiple logistic regression model predicting social, clinical, and laboratory risk factors of MCI-to-dementia progression.

Parameter	Regression coefficient: log odds (standard error)	OR (95% CI)	*z*-score	*p*-value
Intercept	−19.8 (11.1)	–	−1.78	7.5 × 10^−2^
Age, years	0.17 (0.12)	1.19 (0.94–1.5)	1.4	1.6 × 10^−2^
Sex, men	0.42 (0.62)	1.52 (0.45–5.13)	0.67	5.0 × 10^−2^
Follow-up period, years	0.76 (0.57)	2.14 (0.7–6.54)	1.34	1.8 × 10^−2^
Depression	1.03 (0.49)	2.8 (1.07–7.32)	2.09	3.7 × 10^−2^
Total cholesterol, mm/L	0.48 (0.21)	1.62 (1.07–2.44)	2.27	2.3 × 10^−2^
Hobby	−1.42 (0.50)	0.24 (0.09–0.64)	−2.84	4.5 × 10^−3^

OR, odds ratio; CI, confidence interval.

### 3.2 Genome-wide association study of CR in long-living adults

**Figure 3:**
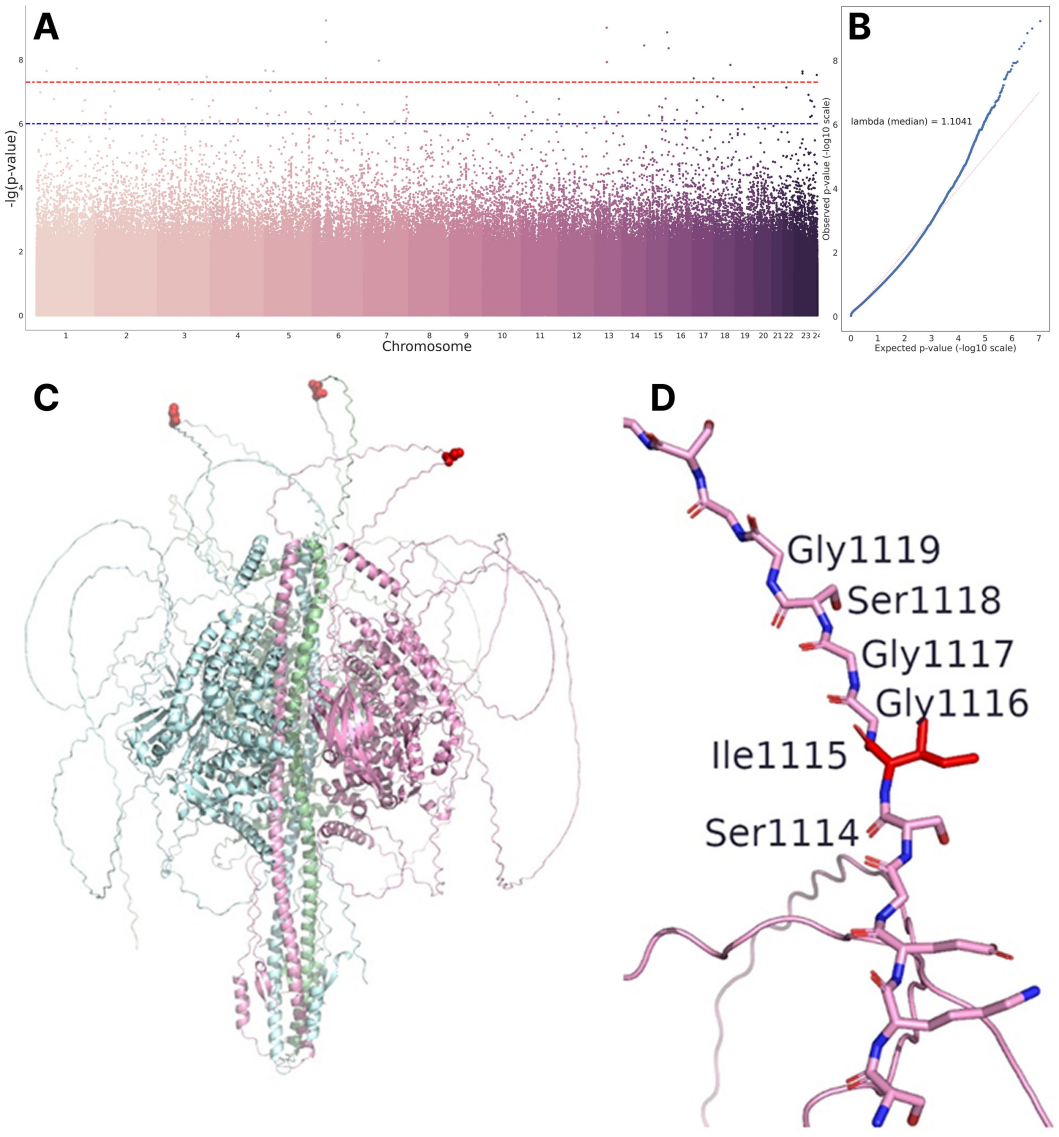
**(A)** Manhattan plot representing GWAS results. **(B)** QQ-plot for GWAS results. **(C)** SynGAP trimer obtained using AlphaFold3. The red spheres indicate the position of Ile1115. **(D)** Ile1115 in the loop region.

Significant associations are presented in Table 3 and illustrated in Figures 3A,B. We identified 22 significant polymorphisms in the *LOC102723446, NR2F2-AS1, LOC105370228, RAP1GAP2, LOC107986537, DIAPH3, TDRD3, SYNGAP1, SYNGAP1-AS1,* and *RPTOR* genes, in non-coding RNAs (*LINC01344, LINC01924,* and *NR2F2-AS1*), pseudogenes (*GGNBP1* and *PRKXP1*), and intergenic regions.

**Table 3:** Single nucleotide polymorphisms significantly associated with CR after cross-validation.

Chromosome, position, substitution	Regression coefficient	*p*-value	Gene	Variant type	dbSNP ID	Within TF binding site*
chr7:45979433: G: A	−32.28	1.07 × 10^−08^	*LOC102723446*	Intronic	rs150184078	*—*
chr15:96181388: G: A	−35.08	1.38 × 10^−09^	*NR2F2-AS1*	Intronic	rs137961036	*POLR2A, ZNF629, SPI1, ZMYM3, BCOR, KMD1A*
chr5:1868550: A: G	−32.24	2.16 × 10^−08^		Intergenic	rs114530160	*EZH2*
chr13:60380804: A: G	−32.47	1.18 × 10^−08^	*LOC105370228*	Intronic	rs116878859	*—*
chr1:37130857: G: T	−18.17	2.23 × 10^−08^		intergenic	rs115709053	*CTCF, RAD21*
chr23:45389981: A: G	−24.93	2.68 × 10^−08^		Intergenic	rs149259839	*—*
chr23:45447047: C: G	−27.86	2.31 × 10^−08^		Intergenic	rs139146816	*—*
chr13:59551403: C: CA	−32.49	9.88 × 10^−10^		Intergenic		*—*
chr6:33562851: T: TTGTGTG	−29.63	3.78 × 10^−08^	*GGNBP1*	Intronic		*—*
chr15:100556786: C: T	−35.02	4.34 × 10^−09^	*PRKXP1*	Intronic	rs200843758	*—*
chr17:3031361: A: T	−32.64	3.82 × 10^−08^	*RAP1GAP2*	Intronic	rs7221569	*—*
chr6:33523156: C: G	−32.23	2.77 × 10^−09^	*LOC107986537*	Intronic	rs146134558	*—*
chr1:182298909: C: T	−32.46	1.86 × 10^−08^	*LINC01344*	Intronic	rs191770718	*ZNF12, GABPA, RBFOX2*
chr18:64351846: C: G	−34.39	1.45 × 10^−08^	*LINC01924*	Intronic	rs79231290	*—*
chr3:184624554: A: G	−32.96	3.43 × 10^−08^		Intergenic	rs551037526	*—*
chr13:60088534: G: A	−32.47	1.18 × 10^−08^	*DIAPH3*	Intronic	rs150254300	*—*
chr23:151262965: A: G	−4.99	2.98 × 10^−08^		Intergenic	rs192721259	*—*
chr13:60534171: G: A	−32.47	1.18 × 10^−08^	*TDRD3*	Intronic	rs181605481	*—*
chr6:33443896: T: C	−31.71	5.84 × 10^−10^	*SYNGAP1, SYNGAP1-AS1*	Missense	rs191549504	*—*
chr17:80905429: CA: C	−33.19	3.85 × 10^−08^	*RPTOR*	intronic		*—*
chr5:28803147: C: A	−31.45	2.27 × 10^−08^		Intergenic	rs1161008264	*—*
chr14:95971120: A: ATTTTT	−34.2	3.57 × 10^−09^		Intergenic		*—*

*Annotation of SNP within transcription factor (TF) binding sites using Factorbook (Pratt et al., 2022).

Most of the variants listed above are located in intronic and intergenic regions of the genome and may only have regulatory effects. However, we found a missense variant in the *SYNGAP1* gene, resulting from the isoleucine-to-threonine replacement at position 1,115 (National Center for Biotechnology Information, 2024a). Isoleucine is a non-polar, highly hydrophobic aliphatic amino acid, while threonine becomes polar and hydrophilic due to the presence of a hydroxyl group. This radical amino acid replacement should lead to structural and conformational changes in the protein. To test this hypothesis, a full-length model of the SynGAP protein was obtained using the AlphaFold3 algorithm (Figure 3C). However, position 1,115 is located in a region lacking a stable secondary structure, making it difficult to unambiguously describe the effect of the substitution.

Moreover, the region of the protein containing this position consists of polar amino acids and glycine (e.g., amino acid sequence 1,110–1,130 looks as follows: SKEGSIGGSGGGGGGGGGGGLK, Figure 3D). Thus, threonine at position 1,115 is unlikely to cause significant conformational changes, since polar threonine is surrounded by other polar amino acids. There is currently no data on the involvement of this position in protein–protein interactions. The lack of data, along with the low confidence level of AlphaFold3 in the structure of the protein domain under consideration, and the biophysical annotation of the Ile1115Thr replacement poses a significant challenge.

Additionally, we studied 68 sub-significant polymorphisms, i.e., with an average *p*-value of below 5 × 10^−7^ but over the genome-wide significance threshold of 5 × 10^−8^. These polymorphisms are presented in Supplementary Table S4. A significant number of these polymorphisms were also located in intergenic regions or in genes, the biological functions of which have yet to be established. The findings in genes with known biological functions, such as *KRT16* (a missense variant), *NFASC, SV2B, MARCHF1,* and *ERC1* (intronic variants) are of particular interest, since they may indicate potential biological mechanisms underlying CR in long-living individuals.

To assess the cumulative contribution of polymorphisms to the dynamics of cognitive impairment, as measured by CR, a linear model was built using a set of significant non-genetic parameters and polymorphisms that surpassed the significance threshold of 5 × 10^−7^ but—potentially due to the insufficient sample size—did not reach the genome-wide significance threshold of 5 × 10^−8^. The model was adjusted for sex and age.

The final formula was as follows:


$$\mathrm{CR}=48,47+\beta_{g}G+\beta_{c}C$$

where $\beta_{g},\beta_{c}$ are the coefficients for the matrix of the number of risk alleles (G) and the matrix of covariates (C).

A regularization coefficient of λ=10 was applied during loss function training. The model’s predictive performance was assessed using the coefficient of determination R2 = 0.348 on the training set and R2 = 0.017 on the test set. Supplementary Table S5 provides all regression coefficients.

## 4 Discussion

Aging is an independent factor contributing to neuromorphological changes in the central nervous system. Certain age-related processes, such as cortical atrophy, amyloid plaques, neurofibrillary tangles, and Lewy body accumulation, are common in older individuals and may be considered normal aging phenomena rather than signs of specific diseases (Wyss-Coray, 2016). Our study examined long-lived adults (90 + years) without dementia or diagnosed neurodegenerative disorders, all receiving cardiovascular medications and experiencing no acute cerebrovascular events during the follow-up. This carefully selected cohort allowed us to evaluate factors influencing cognitive impairment progression while controlling for neuromorphological changes associated with aging.

While our findings generally support existing research on CR, most previous studies focused on younger elderly populations (60–90 years), often excluding or underrepresenting long-living adults. Emerging evidence suggests that CR mechanisms in extreme longevity may differ from those in younger elderly groups (Kawas, 2008). This highlights the importance of age-specific approaches when studying cognitive aging trajectories.

### 4.1 Clinical and lifestyle predictors of CR

Consistent with broader literature (Silva et al., 2019), we found that dyslipidemia (particularly elevated cholesterol levels) and glucose levels were persistent risk factors for cognitive decline across age groups. Many studies suggest that the effect of hypertension on cognitive status is more pronounced in middle-aged individuals and tends to diminish in older adults (Staessen et al., 2007). We did not find an association between blood pressure levels and CR.

BMI demonstrated varying associations with the risk of dementia across different age groups: in middle-aged individuals, higher BMI increased the risk, while in older adults (≥65 years) it showed a protective effect, in line with previous observations (Fitzpatrick et al., 2009), and contributed to greater CR. However, most of our participants were not clinically obese. Our findings support the established protective factors, including physical activity (mobility/walking), preserved hearing, and pet ownership (Livingston et al., 2020; Erema et al., 2024). Notably, we identified chronic anemia as a risk factor for cognitive decline in extreme longevity. While hemic hypoxia’s neurological effects are well-documented, its significant association in long-living adults—but not younger groups—highlights both the vulnerability of aging brain tissue and a clinically modifiable target for intervention.

While education and socioeconomic factors are recognized predictors of CR predictors in younger populations (Xu et al., 2015), our study revealed different patterns in long-living adults. We found no association between educational attainment and cognitive outcomes, consistent with other longevity studies (Kawas and Corrada, 2006; Kawas, 2008). Meanwhile, psychological wellbeing—higher quality of life, absence of depression, and hobbies—significantly protected against cognitive decline.

### 4.2 Genetic and molecular mechanisms of CR

Although the APOE ε4 allele is an established risk factor for Alzheimer’s in younger elderly populations, we found no significant associations between this allele and cognitive decline or MCI-to-dementia progression in long-living adults. This suggests that by the age of 90, other factors may outweigh the contribution of the APOE ε4 allele to the pathogenesis of Alzheimer’s disease. Our genetic analysis identified several factors potentially influencing CR, including understudied genes (*LOC102723446* and *LOC105370228*, intestinal expression; *LOC107986537*, cardiac expression) and functionally significant intronic variants in *RAP1GAP2* (regulating platelet aggregation via Rap1 pathways) (Schultess et al., 2005), *DIAPH3* (involved in actin cytoskeleton organization and linked to auditory neuropathy) (Sánchez-Martínez et al., 2017), and *RPTOR* (a key mTORC1 component mediating nutrient/growth responses expressing in various tissues). These findings suggest potential roles for vascular (*RAP1GAP2*), structural (*DIAPH3*), and metabolic (*RPTOR*) pathways in maintaining cognitive function in extremely advanced age. Moreover, some variants are located within transcription factor binding sites, which may provide them mechanisms whereby they influence the expression of their corresponding genes.

We identified a significant association between CR and a missense variant (Ile1115Thr, rs191549504) in *SYNGAP1*, which encodes a postsynaptic protein crucial for glutamatergic signaling in the forebrain and hippocampus (Sheng and Kim, 2011; The Human Protein Atlas, 2024). The protein contains calcium-binding regions and a GAP domain, which activates GTPases from the Ras and Rap (Pena et al., 2008). While SYNGAP1 is known for its roles in neurodevelopment and rare neurodevelopmental disorders (Agarwal et al., 2019), the detected variant, which is classified as benign in ClinVar (National Center for Biotechnology Information, 2024b), may represent a novel modifier of cognitive aging, particularly as it overlaps with the antisense RNA *SYNGAP1-AS1*, suggesting potential regulatory effects. However, the novelty of this finding warrants cautious interpretation and requires independent validation in other cohorts to confirm its association with cognitive resilience in aging.

We found a sub-significant missense variant in the Keratin-16 (*KRT16*) gene, neurobiological implications of which are difficult to interpret. We also found sub-significant polymorphisms in genes involved in neurophysiology, such as *NFASC, SV2, MARCHF1,* and *ERC1*. Neurofascin (NFASC) plays a role in the growth and convergence of nerve processes, as well as the organization of initial axon segments and nodes of Ranvier on axons during early development. This protein links the extracellular matrix and the axonal cytoskeleton. Synaptic vesicle protein 2 (SV2) is localized in synaptic vesicles and is involved in the regulation of their transport and exocytosis. MARCHF1 is a member of an E3 ubiquitin ligase family. The *ERC1* gene encodes a protein from a family of RIM-binding proteins, which are active zone proteins regulating the release of neurotransmitters (National Center for Biotechnology Information, 2024a).

Thus, our analysis revealed variants in genes that may be involved in neuroplasticity and the clearance of misfolded proteins, which, after the age of 90, may affect the brain’s resilience to changes that are unrelated to existing diseases and conditions. A significant number of findings were located in non-coding regions of the genome, as well as in genes with poorly known functions, suggesting that there may be numerous unidentified pathways involved in these changes, including regulatory genomic regions.

The polygenic risk model incorporating CR-associated genetic variants showed limited predictive performance, attributable to the small sample size and the cohort’s sex composition, reflecting longer lifespan among women. Conducting a prospective study in a large cohort of long-living adults presents inherent difficulties due to their small number and high mortality rates, resulting in significant participant attrition. Thus, our findings underscore that cognitive resilience is a multifactorial phenomenon, not solely attributable to genetics. Incorporating additional data modalities, such as neuroimaging (e.g., MRI to assess brain structure and integrity) and laboratory biomarkers, could provide a more holistic perspective.

### 4.3 Limitations

The generalizability of our findings may be limited by a healthy participant selection bias, inherent in studies of long-living adults due to the exclusion of those with severe debilitating conditions. The reliance on the MMSE as the primary cognitive assessment tool may constitute another limitation of this study. Furthermore, the lack of an assessment of activities of daily living limits our ability to more precisely evaluate the degree of functional autonomy loss. Although widely used, the MMSE has limited sensitivity for detecting mild cognitive impairment and assessing high cognitive function. The use of an unconventional metric for assessing cognitive resilience based on the percentage change in MMSE may not fully capture variance in cognitive trajectories. Moreover, our eligibility criteria solely based on the objective MMSE threshold may have overlooked subjective cognitive complaints. Additional limitations include the lack of data on medication use, a potential confounder that was not controlled for, and the absence of neuroimaging and neuropathological data, preventing the validation of our model against objective biological markers of brain aging and neurodegeneration. Despite these limitations, the findings of this study advance our understanding of the sociodemographic, clinical, and genetic factors involved in CR and highlight the need for further research integrating genetic and non-genetic factors.

## Data Availability

The original contributions presented in the study are included in the article/Supplementary material, further inquiries can be directed to the corresponding author.
