# Supplementary material for: Clinical, social, molecular, and genetic predictors of cognitive resilience in long-living adults without dementia
Source: Front Dement. 2026 Feb 16;4:1699695. doi: 10.3389/frdem.2025.1699695 (PMC12950572; doi:10.3389/frdem.2025.1699695)
Supplement: Supplementary file 1 [file Supplementary_file_1.docx]

**Supplementary materials**


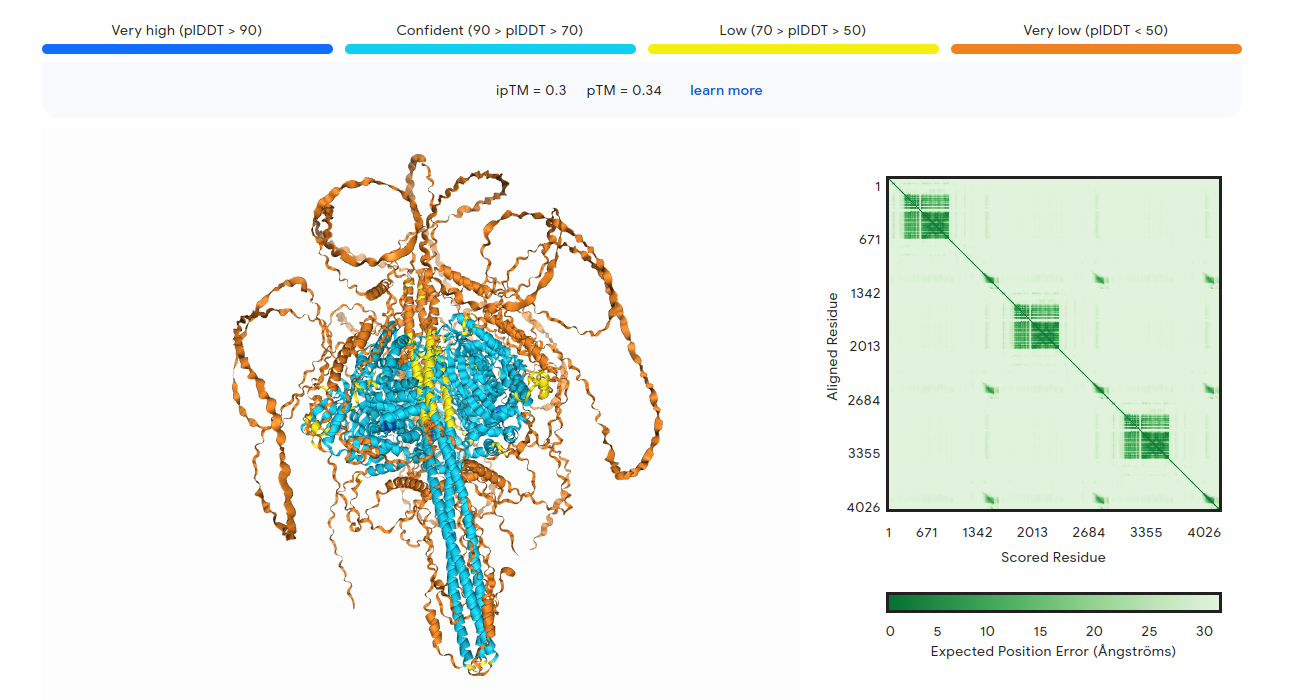


**Figure S1.** Results obtained with the AlphaFOLD3 algorithm: a 3D structure indicating the predictive quality.

**Table S1.** General characteristics of the study cohort. Qualitative variables are given as medians and 1 and 3 interquartile (Me (Q1, Q3)); nominal variables, as absolute frequencies of occurrence and percentages (%).

| Parameter | | | N | All-participants,  N = 295 | Women,  N = 243 | Men,  N = 52 | Value |
| --- | --- | --- | --- | --- | --- | --- | --- |
| Sociodemographic Parameters | | | | | | | |
| Age, years | | | 295 | 92.00 (90.00; 93.00) | 92.00 (90.00; 93.00) | 91.00 (90.00; 93.00) | 3.2×10^-1^ |
| Follow-up period, years | | | 295 | 2.01 (1.78; 2.43) | 1.99 (1.81; 2.41) | 2.05 (1.72; 2.57) | 7.6×10^-1^ |
| Educational attainment | | *Secondary or lower* | 287 | 79 (28%) | 73 (31%) | 6 (12%) | **2.5×10^-2^** |
|  |  | *Secondary and vocational* |  | 81 (28%) | 66 (28%) | 15 (31%) |  |
|  |  | *Higher, including academic degrees* |  | 127 (44%) | 99 (42%) | 28 (57%) |  |
| Life-long occupation | | *Mental and manual labor* | 292 | 108 (37%) | 84 (35%) | 24 (47%) | 8.1×10^-2^ |
|  |  | *Mental labor* |  | 127 (43%) | 112 (46%) | 15 (29%) |  |
|  |  | *Manual labor* |  | 57 (20%) | 45 (19%) | 12 (24%) |  |
| Life-long income level | | *Low* | 274 | 18 (6,6%) | 17 (7,5%) | 1 (2,1%) | 2,4×10^-1^ |
|  |  | *Medium* |  | 228 (83%) | 189 (83%) | 39 (83%) |  |
|  |  | *High* |  | 28 (10%) | 21 (9.3%) | 7 (15%) |  |
| Living arrangements | | *Alone* | 295 | 144 (49%) | 113 (47%) | 31 (60%) | 1.1×10^-1^ |
|  |  | *With family members* |  | 118 (40%) | 104 (43%) | 14 (27%) |  |
|  |  | *In nursing home* |  | 33 (11%) | 26 (11%) | 7 (13%) |  |
| Currently working | | | 295 | 3 (1,0%) | 2 (0.8%) | 1 (1.9%) | 4.8×10^-1^ |
| Age at retirement, years | | | 281 | 63 (57; 70) | 61 (55; 70) | 70 (60; 71) | **3.1×10^-4^** |
| Smoking status | | *Current smoker* | 274 | 28 (10%) | 21 (9.3%) | 7 (15%) | 2.3×10^-1^ |
|  |  | *Former smoker* |  | 228 (83%) | 189 (83%) | 39 (83%) |  |
|  |  | *Never-smoker* |  | 18 (6.6%) | 17 (7.5%) | 1 (2.1%) |  |
| Having hobbies | | | 267 | 94 (35%) | 80 (36%) | 14 (30%) | 4.5×10^-1^ |
| Social interactions | *None in the last year* | | 288 | 10 (3.5%) | 5 (2.1%) | 5 (10%) | **4.2×10^-2^** |
|  | *1-2 times a year* | |  | 0 (0%) | 0 (0%) | 0 (0%) |  |
|  | *1-2 a month* | |  | 30 (10%) | 25 (11%) | 5 (10%) |  |
|  | *1-2 a week* | |  | 115 (40%) | 93 (39%) | 22 (44%) |  |
|  | *Daily* | |  | 133 (46%) | 115 (48%) | 18 (36%) |  |
| Neuropsychological Profile | | | | | | | |
| Baseline MMSE score | | | 295 | 26 (24; 28) | 26 (24; 28) | 25 (24; 27.5) | 7.5×10^-1^ |
| Amnestic cognitive impairment | | | 295 | 43 (15%) | 36 (15%) | 7 (13%) | 8×10^-1^ |
| Follow-up MMSE score | | | 295 | 26 (22; 28) | 26 (21; 28) | 26 (22; 28) | 5.2×10^-1^ |
| 5-item GDS score | | | 295 | 1 (0; 2) | 1 (0; 2) | 1 (0; 2) | 1.1×10^-1^ |
| Subjective quality of life | | | 280 | 7 (5; 8) | 7 (5; 8) | 7 (6; 8) | 5.9×10^-1^ |
| Health Status & Comorbidities | | | | | | | |
| BMI | | | 266 | 26.1 (23.7; 29) | 26.2 (23.7; 29) | 25.6 (23.9;29) | 6.9×10^-1^ |
| Hemodynamically significant carotid atherosclerosis | | | 292 | 37 (13%) | 29 (12%) | 8 (15%) | 5.2×10^-1^ |
| Polypharmacy | | | 283 | 133 (47%) | 103 (44%) | 30 (60%) | **4.2×10^-2^** |
| Urinary incontinence | | | 295 | 174 (59%) | 152 (63%) | 22 (42%) | **7.2×10^-3^** |
| Mobility | | *Living home* | 295 | 203 (69%) | 161 (66%) | 42 (81%) | 1.3×10^-1^ |
|  |  | *Moving indoors* |  | 81 (27%) | 72 (30%) | 9 (17%) |  |
|  |  | *Bedridden/chairbound* |  | 11 (3.7%) | 10 (4.1%) | 1 (1.9%) |  |
| Chronic pain | | | 295 | 185 (63%) | 160 (66%) | 25 (48%) | **1.6×10^-2^** |
| Arterial hypertension | | | 294 | 274 (93%) | 227 (94%) | 47 (90%) | 3.7×10^-1^ |
| Prior ACI | | | 295 | 47 (16%) | 41 (17%) | 6 (12%) | 3.4×10^-1^ |
| Type 2 diabetes | | | 293 | 42 (14%) | 37 (15%) | 5 (9.6%) | 2.8×10^-1^ |
| Congestive heart failure | | | 294 | 187 (64%) | 151 (62%) | 36 (69%) | 3.5×10^-1^ |
| Chronic anemia | | | 294 | 43 (15%) | 36 (15%) | 7 (13%) | 7.9×10^-1^ |
| Chronic kidney disease | | | 294 | 15 (5.1%) | 12 (5.0%) | 3 (5.8%) | 7.3×10^-1^ |
| Metabolic syndrome | | | 295 | 71 (24%) | 61 (25%) | 10 (19%) | 3.7×10^-1^ |

**Table S2.** Linear regression results for CR as a dependable variable, obtained using different predictors and the same covariates: age, sex, and initial MMSE score. Each line presents induvial parameters of each single-predictor model.

| Model parameter | | | Regression coefficient, β | Standard error, SE | t-value | p-value |
| --- | --- | --- | --- | --- | --- | --- |
| Sociodemographic Predictors | | | | | | |
| Sex, men | | | 0.92 | 1.57 | 0.59 | 0.559 |
| Age | | | -1.32 | 0.33 | -4.05 | **7.4×10^-5^** |
| Mobility | *Moving indoors vs relatively unrestricted* | | -4.05 | 1.44 | -2.82 | **5.3×10^-3^** |
|  | *Bedridden vs relatively unrestricted* | | -10.27 | 3.35 | -3.07 | **2.5×10^-3^** |
| Educational attainment | *Secondary and lower vs higher* | | -2.48 | 1.59 | -1.57 | 0.119 |
|  | *Secondary and vocational vs higher* | | -1.88 | 1.48 | -1.28 | 0.204 |
| Lifelong income level | *Low vs high* | | -5.18 | 3.13 | -1.66 | 0.099 |
|  | *Medium vs high* | | -2.83 | 2.1 | -1.35 | 0.178 |
| Living arrangements | *With family members vs alone* | | -1.5 | 1.28 | -1.17 | 0.245 |
|  | *In nursing home vs alone* | | -1.18 | 1.94 | -0.61 | 0.544 |
| Going on walks | *Non vs daily* | | -5.83 | 1.59 | -3.66 | **3.3×10^-4^** |
|  | *Several times a week vs daily* | | -1.6 | 1.97 | -0.81 | 0,417 |
|  | *Once a week vs daily* | | -4.35 | 2.19 | -1.98 | 4.9×10^-2^ |
|  | *Less than once a week vs daily* | | -0.04 | 2.38 | -0.02 | 0,987 |
| Smoking status | *Never-smoker vs current smoker* | | -5.13 | 2.89 | -1.78 | 0,078 |
|  | *Former smoker vs current smoker* | | -2.46 | 1.88 | -1.31 | 0,193 |
| Having hobbies | | | 2.14 | 1.26 | 1.7 | 0.09 |
| Pet ownership | | | -3.33 | 1.6 | -2.08 | **3.9×10^-2^** |
| Retirement duration | | | -0.07 | 0.07 | -1.06 | 0.292 |
| Lifelong occupation type | | *Mental vs metal and manual* | 0.22 | 1.32 | 0.17 | 0,865 |
|  |  | *Manual vs mental and manual* | -2.62 | 1.93 | -1.36 | 0,176 |
| Subjective quality of life score | | | 0.87 | 0.31 | 2.8 | **5.7×10^-3^** |
| Clinical Predictors | | | | | | |
| Hearing acuity | *Marginally lower vs significantly lower* | | 4.48 | 1.59 | 2.82 | **5.4×10^-4^** |
|  | *High vs significantly lower* | | 3.32 | 1.8 | 1.84 | 6.8×10^-2^ |
| Depression | | | -2.83 | 1.23 | -2.31 | **2.2×10^-2^** |
| BMI | | | 0.44 | 0.16 | 2.75 | **6.6×10^-3^** |
| Hemodynamically significant carotid atherosclerosis | | | -0.99 | 1.61 | -0.61 | 0.54 |
| Visual acuity | | | -0.32 | 1.61 | -0.2 | 0.843 |
| Chronic pain | | | 0.01 | 1.25 | 0.008 | 0.994 |
| Type 2 diabetes | | | -1.05 | 1.75 | -0.6 | 0.549 |
| COPD | | | -1.05 | 1.75 | -0.6 | 0.549 |
| Congestive heart failure | | | 1 | 1.23 | 0.82 | 0.416 |
| Atrial fibrillation of any type форма) | | | 0.28 | 1.29 | 0.22 | 0.825 |
| Chronic kidney disease | | | 2.32 | 1.34 | -1.73 | 8.6×10^-2^ |
| Chronic anemia | | | -3.65 | 1.72 | -2.13 | **3.5×10^-2^** |
| Metabolic syndrome | | | 0.65 | 1.37 | 0.47 | 0.639 |
| Biochemical Predictors | | | | | | |
| Glucose, mmol/L | | | -0.96 | 0.47 | -2.02 | **4.5×10^-2^** |
| Total cholesterol, mmol/L | | | -1.01 | 0.51 | -1.98 | **4.9×10^-2^** |
| Total protein, g/L | | | 0.18 | 0.11 | 1.66 | 9.8×10^-2^ |
| Vitamin D, ng/mL | | | 0.22 | 0.37 | 0.58 | 0.561 |
| TSH, mU/L | | | -0.54 | 0.28 | -1.9 | 5.9×10^-2^ |
| *Note*: COPD: chronic obstructive pulmonary disease; TSH: thyroid stimulating hormone. | | | | | | |

**Table S3.** Logistic regression results for developing dementia during the follow-up period as a dependable variable, obtained using different predictors and the same covariates: age, sex, initial MMSE score. Each line presents induvial parameters of each single-predictor model

| Model parameter | | Regression coefficient, β | Standard error, SE | OR (95% CI) | z-score | p-value |
| --- | --- | --- | --- | --- | --- | --- |
| Sociodemographic Predictors | | | | | | |
| Sex, men | | 0.01 | 0.52 | 1.01 (0.36-2.8) | 0.03 | 0.979 |
| Age, years | | 0.24 | 0.11 | 1.27 (1.02-1.58) | 2.14 | **3.3×10^-2^** |
| Follow-up period, years | | 0.56 | 0.33 | 1.75 (0.92-3.34) | 1.66 | 9.7×10^-2^ |
| Mobility | *Moving indoors vs relatively unrestricted* | 0.87 | 0.46 | 2.39 (0.97-5.88) | 1.9 | 5.8×10^-2^ |
|  | *Bedridden vs relatively unrestricted* | 2.22 | 1.14 | 9.21 (0.99-86) | 1.95 | 5.1×10^-2^ |
| Educational attainment | *Secondary and lower vs higher* | 0.56 | 0.5 | 1.75 (0.66-4.66) | 1.14 | 0,256 |
|  | *Secondary and vocational vs higher* | 0.35 | 0.52 | 1.42 (0.51-3.93) | 0.68 | 0,495 |
| Lifelong income level | *Low vs high* | 1.88 | 1.21 | 6.55 (0.61-70.22) | 1.56 | 0,119 |
|  | *Medium vs high* | 2.1 | 1.6 | 8.17 (0.35-187.92) | 1.1 | 0,631 |
| Living arrangements | *With family members vs alone* | 0.87 | 0.44 | 2.39 (1.01-5.65) | 2.01 | **4.5×10^-2^** |
|  | *In nursing home vs alone* | 0.09 | 0.72 | 1.09 (0.27-4.49) | 0.13 | 0,9 |
| Lifelong occupation type | *Mental vs metal and manual* | -0.12 | 0.43 | 0.89 (0.38-2.06) | -0.27 | 0.787 |
|  | *Manual vs mental and manual* | -0.12 | 0.58 | 0.89 (0.28-2.76) | -0.2 | 0.839 |
| Going on walks | *Non vs daily* | 0.63 | 0.52 | 1.88 (0.68-5.2) | 1.22 | 0,223 |
|  | *Several times a week vs daily* | -0.23 | 0.62 | 0.79 (0.24-2.68) | -0.37 | 0,714 |
|  | *One a week vs daily* | -0.64 | 0.71 | 0.53 (0.13-2.12) | -0.9 | 0,368 |
|  | *Less than once a week vs daily* | -0.25 | 0.78 | 0.78 (0.17-3.59) | -0.32 | 0,749 |
| Smoking status | *Never-smoker vs current smoker* | 1.88 | 1.21 | 6.55 (0.61-70.22) | 1.56 | 0,119 |
|  | *Former smoker vs current smoker* | 2.1 | 1.6 | 8.17 (0.35-187.92) | 1.1 | 0,631 |
| Having hobbies | | -1.03 | 0.45 | 0.36 (0.15-0.86) | -2.27 | **2.4×10^-2^** |
| Pet ownership | | 0.46 | 0.57 | 1.58 (0.52-4.84) | 0.81 | 0.418 |
| Retirement duration | | 0.02 | 0.02 | 1.02 (0.98-1.06) | 0.65 | 0.516 |
| Subjective quality of life score | | 0.86 | 0.41 | 2.36 (1.06-5.28) | 2.13 | **3.4×10^-2^** |
| Clinical Predictors | | | | | | |
| Hearing acuity | *Marginally lower vs significantly lower* | -0.22 | 0.48 | 0.8 (0.31-2.06) | -0.45 | 0,652 |
|  | *High vs significantly lower* | -0.67 | 0.64 | 0.51 (0.15-1.79) | -1.04 | 0,299 |
| BMI | | -0.34 | 0.12 | 0.71 (0.56-0.9) | -2.8 | **5.1×10^-3^** |
| Hemodynamically significant carotid atherosclerosis | | -0.06 | 0.06 | 0.94 (0.84-1.06) | -1.03 | 0.305 |
| Visual acuity | | 0.25 | 0.55 | 1.28 (0.44-3.77) | 0.45 | 0.653 |
| Subjective quality of life score | | 0.28 | 0.62 | 1.32 (0.39-4.46) | 0.46 | 0.648 |
| Chronic pain | | 0.48 | 0.4 | 1.62 (0.74-3.54) | 1.184 | 0.236 |
| Type 2 diabetes | | 0.09 | 0.56 | 1.09 (0.37-3.28) | 0.16 | 0.874 |
| COPD | | 0.09 | 0.56 | 1.09 (0.37-3.28) | 0.16 | 0.874 |
| Congestive heart failure | | -0.26 | 0.42 | 0.77 (0.34-1.76) | -0.62 | 0.538 |
| Atrial fibrillation of any type форма) | | -0.33 | 0.43 | 0.72 (0.31-1.67) | -0.77 | 0.443 |
| Chronic kidney disease | | 0.44 | 0.46 | 1.55 (0.63-3.83) | 0.96 | 0.336 |
| Chronic anemia | | 0.63 | 0.49 | 1.88 (0.72-4.91) | 1.31 | 0.191 |
| Metabolic syndrome | | -0.05 | 0.45 | 0.95 (0.39-2.3) | -0.12 | 0.903 |
| Biochemical Predictors | | | | | | |
| Glucose, mmol/L | | 0.16 | 0.15 | 1.17 (0.87-1.57) | 1.07 | 0.287 |
| Total cholesterol, mmol/L | | 0.44 | 0.18 | 1.55 (1.09-2.21) | 2.52 | **1.2×10^-2^** |
| Total protein, g/L | | -0.02 | 0.04 | 0.98 (0.91-1.06) | -0.67 | 0.503 |
| Vitamin D, ng/mL | | 0.01 | 0.02 | 1.01 (0.97-1.05) | 0.83 | 0.405 |
| TSH, mU/L | | 0.05 | 0.08 | 1.05 (0.9-1.23) | 0.69 | 0.488 |
| *Note*: COPD: chronic obstructive pulmonary disease; TSH: thyroid stimulating hormone. | | | | | | |

**Table S4.** Annotation of sub-significant variants from the GWAS after cross-validation (5×10^-8^ < p-value < 5×10^-7^)

| Chromosome, position, substitution | Average regression coefficient | Gene | Variant | dbSNP ID |
| --- | --- | --- | --- | --- |
| 7:157106412:T:C | -25.7058547 |  | intergene | rs569569899 |
| 17:13302062:C:CA | -30.3162446 | LINC02093 | intron | rs1005403785 |
| 19:19939590:TCTC:T | -31.5055495 |  | intergene |  |
| 2:36066110:G:A | -29.7819002 |  | intergene | rs184210911 |
| 11:15375210:CT:C | -30.0528726 |  | intergene | rs940956791 |
| 23:119142370:C:T | -30.1303027 |  | intergene |  |
| 17:41610359:G:A | -27.4806129 | KRT16 | missense | rs56259134 |
| 1:204941695:G:A | -29.7341056 | NFASC | intron | rs189546335 |
| 17:41609137:G:A | -27.4806129 |  | intergene | rs148689045 |
| 2:52063830:G:C | -29.378687 | LOC730100 | intron | rs148083714 |
| 2:35998413:A:T | -29.7819002 |  | intergene | rs77821055 |
| 7:152397700:T:A | -26.9001371 | KMT2C | intron | rs118001704 |
| 3:4032910:G:T | -24.1473348 | LOC102723512 | intron | rs115744699 |
| 5:7363439:A:C | -29.7809117 | LOC105374645 | intron | rs138493481 |
| 5:18319699:C:A | -28.9333568 |  | intergene | rs114354631 |
| 1:215184472:C:T | -31.0215269 | KCNK2 | intron | rs114786557 |
| 4:164007630:T:TTCTC | -29.7658444 | MARCHF1 | intron |  |
| 15:66154698:G:A | -29.3391451 | MEGF11 | intron | rs147399175 |
| 6:151386390:C:T | -30.6030449 | ZBTB2 | intron | rs150538036 |
| 10:52655540:G:T | -31.2647019 | LOC105378305 | intron | rs575155296 |
| 5:168643278:C:T | -29.2321001 |  | intergene | rs373976243 |
| 2:52243922:T:C | -29.378687 | LOC730100 | intron | rs77186126 |
| 11:37705631:T:C | -30.3882723 |  | intergene | rs76162401 |
| 1:13602043:AT:A | -31.067219 | PDPN | intron |  |
| 5:18330474:T:C | -28.9333568 |  | intergene | rs74495603 |
| 19:19783511:C:T | -30.9629887 | ZNF56P | intron | rs144855816 |
| 7:152393247:G:A | -26.9001371 | KMT2C | intron | rs184165121 |
| 5:7189401:C:T | -29.7809117 |  | intergene | rs142644161 |
| 7:142137216:T:TA | -30.2384637 | MGAM2 | intron | rs1246258190 |
| 5:7302218:T:G | -29.7809117 | LOC442132 | non coding transcript variant | rs184292649 |
| 16:24961547:G:A | -28.9634024 | ARHGAP17 | intron | rs796631187 |
| 13:100203273:CA:C | -28.8815293 | PCCA | intron | rs1054052209 |
| 10:21874432:TA:T | -29.0726101 | DNAJC1 | intron | rs1336938226 |
| 1:67273684:C:G | -30.7547372 |  | intergene | rs17375360 |
| 6:92314951:T:C | -31.1354483 |  | intergene | rs962806096 |
| 23:136146510:GT:G | -26.6012895 |  | intergene |  |
| 19:56918247:C:A | -29.9324227 |  | intergene | rs112659963 |
| 17:41597852:G:A | -27.4806129 |  | intergene | rs11658200 |
| 7:152486330:T:C | -29.666014 |  | intergene | rs903751141 |
| 18:37782819:T:C | -31.0215269 |  | intergene | rs570237098 |
| 5:7290786:T:C | -29.7809117 |  | intergene | rs187796421 |
| 15:91212978:TA:T | -24.3262723 | SV2B | intron |  |
| 2:52104056:T:G | -29.378687 | LOC124907767 | intron | rs142117787 |
| 17:12213327:C:T | -22.2544701 |  | intergene | rs8078242 |
| 10:120086457:A:T | -27.3178629 |  | intergene | rs11199169 |
| 3:128680357:T:TGGGG | -32.0227507 |  | intergene | rs77120998 |
| 23:108875871:C:A | -27.2132108 |  | intergene |  |
| 23:93350343:T:A | -30.4881103 |  | intergene |  |
| 3:192463244:C:T | -28.1363179 | FGF12 | intron | rs188040612 |
| 17:41587523:C:T | -27.4806129 | KRT14 | 2KB upstream variant | rs3760529 |
| 2:66534326:G:A | -28.059831 | MEIS1 | intron | rs191999528 |
| 12:1484583:C:CT | -31.3554444 | ERC1 | intron | rs1370639481 |
| 1:204944736:G:A | -29.7341056 | NFASC | intron | rs182316082 |
| 1:212309880:A:G | -30.5689423 | PPP2R5A | intron | rs183667209 |
| 15:78663755:C:A | -28.7507819 |  | intergene | rs79640320 |
| 7:152351072:A:G | -29.666014 | KMT2C | intron | rs896911790 |
| 19:20191737:T:G | -31.5055495 | ZNF486 | intron | rs571605401 |
| 7:152371315:A:T | -25.4701585 | KMT2C | intron | rs79682488 |
| 17:12218431:G:A | -22.2544701 |  | intergene | rs151005111 |
| 6:33615670:G:A | -28.1363179 |  | intergene | rs182411527 |
| 6:33377805:A:G | -28.1363179 |  | intergene | rs118109745 |
| 6:170444230:C:T | -29.6614129 | LOC124901477 | intron | rs9460173 |
| 5:78800336:A:AGC | -19.3266761 | ARSB | intron | rs1554071191 |
| 3:73690839:C:CA | -33.9367582 | LOC105377164 | intron | rs77523335 |
| 4:5497053:A:T | -28.9852311 | STK32B | intron | rs562792197 |
| 5:174862312:A:T | -28.9885945 |  | intergene | rs188610595 |
| 22:25266546:G:A | -16.0523581 |  | intergene | rs57911975 |
| 18:10139444:A:G | -30.9762355 |  | intergene | rs138165400 |

**Table S5.** Ridge regression model parameters for CR

| Predictor | Regression coefficient |
| --- | --- |
| Age | 1.2879153 |
| Baseline MMSE score | -0.3173319 |
| BMI | 0.0748204 |
| Glucose | 0.0245716 |
| Total cholesterol | -0.5501741 |
| Hearing acuity (significantly lower) | 0.8940859 |
| Hearing acuity (marginally lower) | -2.2696848 |
| Hearing acuity (high) | 1.3755989 |
| Mobility (bedridden) | -0.3733902 |
| Mobility (moving indoors) | -0.7160579 |
| Mobility (relatively unrestricted) | 1.0894481 |
| Going on walks (none) | -0.8853224 |
| Going on walks (less than once a week) | 0.0111323 |
| Going on walks (once a week) | -0.1664671 |
| Going on walks (several times a week) | 0.5891088 |
| Going on walks (daily) | 0.4515484 |
| 14:95971120:A:ATATTTT | -0.7325590 |
| 6:33523156:C:G | -0.2292489 |
| 13:59551403:CA:C | 0.2610209 |
| 14:95971120:A:ATATATT | -0.5819380 |
| 5:78800336:A:AAGC | -1.9390354 |
| 13:100203273:C:CA | -1.5936434 |
| 16:24961547:G:A | -0.4665869 |
| 3:73690839:C:CA | 0.1230983 |
| 15:100556786:C:T | -1.7634612 |
| 23:151262965:A:G | -0.8604742 |
| 15:26230777:G:GCGCA | -0.2775941 |
| 1:227785499:TA:T | -1.0298930 |
| 9:115558964:T:C | 0.1719795 |
| 17:41587523:C:T | -1.0298930 |
| 15:96181388:G:A | -0.5586860 |
| 6:92314951:T:C | -0.4778165 |
| 23:107749178:TAC:T | -0.6811734 |
| 7:152486330:T:C | -1.0578621 |
| 14:95971120:A:ATTTTTT | -0.7146455 |
| 19:20191737:T:G | -1.0578621 |
| 16:14085457:C:CA | -0.6609198 |
| 15:78807154:A:C | -0.0899309 |
| 6:33615670:G:A | -0.1159150 |
| 7:150033656:T:A | -0.1789034 |
| 5:168643278:C:T | 0.0497803 |
| 5:18330474:T:C | -0.3866027 |
| 11:15375210:CT:C | -1.5651229 |
| 15:78710003:T:C | -0.9342551 |
| 5:7290786:T:C | 0.5125329 |
| 7:104741461:CAA:C | -0.8006050 |
| 1:215184472:C:T | 0.8400317 |
| 23:108875871:C:A | -0.3852902 |
| 13:60088534:G:A | -0.1159150 |
| 6:151386390:C:T | -1.5259462 |
| 15:78643932:G:A | -0.6811734 |
| 12:2791297:C:T | -0.0899309 |
| 12:1484583:C:CTT | -1.7890999 |
| 15:66154698:G:A | -0.0899309 |
| 19:19783511:C:T | -1.2441671 |
| 2:62261845:G:A | -0.6564977 |
| 10:21874432:TA:T | -0.4213888 |
| 17:13302062:CA:C | -0.3629525 |
| 17:12218431:G:A | -0.7143044 |
| 3:128680357:T:TGG | -0.4293997 |
| 6:33377805:A:G | -0.8758208 |
| 4:94542336:T:A | -1.2011953 |
| 6:33443896:T:C | -0.1081556 |
| 17:41609137:G:A | -1.2919518 |
| 1:37130857:G:T | -0.7836085 |
| 13:100203273:CA:C | -1.1484332 |
| 3:194994047:G:C | -0.8526719 |
| 13:58847812:A:G | -0.7437199 |
| 22:25266546:G:A | -0.1531413 |
| 3:17147919:C:T | -0.8442833 |
| 15:26230777:G:GCACA | -0.0899309 |
| 7:152393247:G:A | -0.6254990 |
| 1:227785499:T:TA | -0.2889745 |
| 16:14085457:C:CAA | -0.5586860 |
| 6:66966974:A:G | -1.1507087 |
| 5:7363439:A:C | -0.5791760 |
| 16:76972908:C:T | -1.2931693 |
| 23:118383878:C:CTT | -0.9138107 |
| 5:52734016:CT:C | -0.8968247 |
| 5:7189401:C:T | -1.9313925 |
| 11:15375210:C:CT | -1.7148065 |
| 3:128680357:T:TGGG | -1.4456388 |
| 15:91212978:T:TA | -0.7437199 |
| 7:142137216:TA:T | -1.0320662 |
| 3:4032910:G:T | -0.4685744 |
| 23:136146510:G | 0.1719795 |
| 23:93350343:T:A | -0.2144771 |
| 13:60534171:G:A | -0.9421770 |
| 7:157106412:T:C | -0.4751892 |
| 5:28803147:C:A | -1.0604467 |
| 15:78720660:AACAG:A | -0.1531413 |
| 3:128680357:T:TGGGG | -1.5476795 |
| 1:204941695:G:A | -1.5936434 |
| 17:41597852:G:A | -0.6811734 |
| 4:26664628:CT:C | -1.1507087 |
| 2:52243922:T:C | -0.7952992 |
| 4:94542336:T:TAAATA | -0.4751892 |
| 6:170444230:C:T | -1.1101792 |
| 7:152351072:A:G | -1.5233789 |
| 11:37705631:T:C | -1.0660202 |
| 13:60380804:A:G | -1.0990499 |
| 13:59551403:C:CA | -0.7727431 |
| 14:95971120:A:ATTTTT | -0.5051426 |
| 2:209780557:T:C | -2.0963181 |
| 17:80905429:C:CA | -0.2968244 |
| 23:45447047:C:G | 0.0757849 |
| 18:10139444:A:G | 0.3664024 |
| 1:212309880:A:G | -0.0403573 |
| 4:164007630:T:TTCTC | 0.5527088 |
| 5:1868550:A:G | -2.3232568 |
| 10:120086457:A:T | -1.6108416 |
